# Beyond the “inimitable” Goffman: from “social theory” to social theorizing in a Goffmanesque manner

**DOI:** 10.3389/fsoc.2023.1171087

**Published:** 2023-10-25

**Authors:** David Inglis, Christopher Thorpe

**Affiliations:** ^1^Faculty of Social Sciences, University of Helsinki, Helsinki, Finland; ^2^Department of Sociology, Philosophy and Anthropology, University of Exeter, Exeter, United Kingdom

**Keywords:** Goffman, interaction, sociology, theory, methodology, social, writing, literature

## Abstract

Erving Goffman's status as a great social scientist today seems relatively secure. Many commentators highlight his extraordinary capacities to pinpoint the fine-grained details of human behavior in the “interaction order”. But if Goffman's brilliance in this respect was deeply rooted in his various and interlocking personal, existential, social, and intellectual idiosyncrasies, and his intellectual practice is inimitable, the degree to which anyone else could, or should try to, imitate Goffman's intellectual practice today, remains an open question. This is especially so when we consider that such practice was grounded in notably wide reading across disciplines and in world literature, a highly developed analytical manner that was inseparable from a notable literary talent in composing published texts, and an open-mindedness about the gathering of data sources in ways that some today find methodologically much too promiscuous. The paper initially considers these issues: the multiple “Goffmans” that exegetes and commentators have identified; how such persons have claimed Goffman to be essentially of one or more theoretical persuasions; and how various social theorists have drawn upon Goffman's work. It then moves on to argue that a Goffmanesque kind of social theorizing, is not only possible (if difficult) today, but also vital too. Such theorizing insists on the ongoing role of literary-intellectual and metaphorical ways of thinking and writing, at a time when these are becoming apparently less crucial in studies of human interaction. No matter how technologically advanced such studies may become, they still require some of the intellectual and literary flair that Goffman brought to his scholarly doings. Goffmanesque theorizing can inform new insights into various domains, including the very nature of social change.

## Introduction

It is now more than 40 years since Erving Goffman died. His fame and status as a great social scientist today seem relatively secure, both within sociology and without. Many commentators highlight his extraordinary capacities to pinpoint the fine-grained details of human behavior. Perhaps best known to non-specialists for his analysis of performances of self in everyday life, that was merely one of his various attempts at revealing the patterns within what he dubbed the human “interaction order.”

But if Goffman's brilliance in this respect was deeply rooted in his various and interlocking personal, existential, social, and intellectual idiosyncrasies, the degree to which anyone else could, or should try to, imitate Goffman's intellectual practice today, remains an open question.

This is especially so when we consider that such practice was grounded in notably wide reading across disciplines and in world literature, a highly developed analytical manner that was inseparable from a notable literary talent in composing published texts, and an open-mindedness about the gathering of data sources in ways that some today find methodologically much too promiscuous. Can there today be a *Goffmanesque* form of social science, including a form of social theory, in any meaningful sense of that term (Branaman, 1997), when the man himself was just so particular, if not also in some ways downright peculiar?

This paper argues that a Goffmanesque kind of *social theorizing*, rather than a body of *social theory*, is not only possible (if difficult) today, but also vital too. This is because such theorizing insists on the ongoing role of literary-intellectual and metaphorical ways of thinking and writing—Goffman's core strengths—at a time when these are becoming apparently less crucial in studies of human interaction. No matter how technologically advanced such studies may become, they still require some of the intellectual and literary flair that Goffman possessed.

The paper starts by considering the multiple “Goffmans” that exegetes and commentators have identified. It then considers how such persons have claimed Goffman to be essentially of one or more theoretical persuasions (Psathas, 1996). Then it looks at how various social theorists have drawn upon Goffman's work. Rather than being able to answer any questions as to whether Goffman “really” was a “social theorist” or not (a question famously posed by Giddens, 1988), the latter part of the paper proposes an alternative perspective. It is Goffman's active “theorizing” that is the most valuable component of his intellectual practice today. How he wrote, how he worked on data, how he created insights through metaphor construction, and how he gathered very varied sources of data to work on… All these inseparable activities together make up Goffmanesque intellectual practice. It is these that others might take inspiration from, especially if they think that the social science of human interaction needs creative and literate social theorizing to draw out the deeper significance of what may be implicit in particular data that record the subtle but patterned forms of human interaction.

## Many Goffmans

The generally positive, sometimes hagiographic, tone of writing about Goffman over the last 40 years since his death stands in sharp contrast to the sometimes often hostile assessments of the man and his works during his lifetime (Ditton, 1980). His peers and contemporaries, especially in sociology, frequently held more negative views of Goffman than has become generally the case today (Drew and Wootton, 1988; Treviño, 2003).

The multiple appraisals of Goffman during his lifetime ranged from outright opposition (Gouldner, 1970; Psathas, 1977), through to great admiration, albeit often ambivalent and flecked with caveats and doubts (Dawe, 1973). Writing soon after Goffman's death, Strong (2013; originally published 1983) summarized the widespread reservations about his writing in many quarters. Noting a divide between Goffman's great fame and the apparently minor theoretical and methodological impact of his writings during his lifetime, Strong (2013, p. 146) argued thus:

It is still too easy to dismiss Goffman's main work as amusing, interesting but minor; as applying only to our own society and era, not to other places and times; as the product of a light essayist, not a scientist; as dealing with micro-trivia rather than macrostructure; or, most seriously of all, as fundamentally immoral, as taking a cynical, manipulative, and ultimately destructive view of humanity.

The writings of Goffman draw attention to the various means through which conventional social situations are enabled, enacted, and reproduced (Lemert and Branaman, 1997). A convention that has emerged in writing about Goffman since he died in 1982 has mostly involved paying fulsome tribute to him. The positive claims-making contents found within the spate of eulogies [e.g., Daniels, 1983; Freidson, 1983; Williams, 1983; Bergesen, 1984; Hymes, 1984; Lofland, 1984; Marx, 1984; Collins, 1986; Strong, 2013 (originally published 1983)] published in his honor in the years immediately after his death, have been endlessly reproduced and elaborated on over the last four decades. Many today would agree with the claims made in the obituary tribute written by Daniels (1983): there is little or “no doubt that Goffman changed the way we think about the world we live in and our passage through it. He examined apparently insignificant, unnoticeable, conventional activities and found important social principles embedded in routine.”

Thus, the markedly mixed appreciations of Goffman during his working life have for the most part been replaced by a highly positive posthumous framing, reproduced in waves of texts celebrating his work, while seeking to update it to new scientific and social concerns (e.g., Riggins, 1990; Treviño, 2003; Scheff, 2006; Jacobsen, 2009; Jacobsen and Kristiansen, 2015; Fine, 2018). This encompasses tropes like Goffman-the-innovator and Goffman-the-fearless-interrogator of human foibles, as well as Goffman the author whose works we have still yet barely begun to understand the true significance of (Collins, 1986, p. 111; Scheff, 2003, p. 60). Bitingly critical commentaries on Goffman's work are these days relatively few and thereby unusual (one such is Denzin, 2002).

Conventionalised forms of tribute to Goffman include praising the following sorts of things: his acute eye for picking up on the most telling details in patterns of human interaction (MacIntyre, 1969); his distinctive writing style, which is felt to be simultaneously sharp, elegant, witty, ironic, caustic, stimulating and unnerving (Lemert, 2003); his truly ground-breaking work in setting up the “interaction order” as a site for serious scholarly analysis (Goffman, 1983; Rawls, 1987); his widespread, if diffuse, intellectual legacy, both within his home discipline of sociology—with which he had an often ambivalent set of relations (Rogers, 2003)—and throughout the wider social and human sciences (Menand, 2009). The epithets “Goffmanian” and “Goffmanesque” have come to act as shorthand descriptions of many sorts of performance-related and interactional dynamics. These are terms that continue to resonate throughout many disciplinary and interdisciplinary contexts today (Smith, 1999, p. 6; also Smith, 1988).

Nonetheless, a few cracks exist within the generally highly positive present-day presentations of Goffman. First, the apparent “inimitability” of Goffman provokes some ambivalences of interpretation (Delaney, 2014, p. 88). The assertion of inimitability rests in exegetes stressing the idiosyncrasies of both Goffman the person and Goffman the intellectual producer; the word “quirky” is widely applied to both, as Scheff (2003) complains. The idiosyncrasy applies variously to: his capacities to grasp things other people coming before him could not grasp; to his distinctive research practice—gathering data from every possible source, hither and thither, and then subjecting them to his own unique interpretative abilities; and also to his writing style (brilliant but irreproducible) (Daniels, 1983). If Goffman was so unique as a thinker, writer, and researcher, then perhaps it is simply impossible to imitate him (Fine and Manning, 2003).

This thought is often connected in exegetes' writings to the notion that it is not at all surprising that, despite his obvious superficial influences on many sociologists, there arose no distinctive “Goffman School” during his lifetime or thereafter. One could not, and still cannot, train apprentices directly to follow in Goffman's footsteps, precisely because the pathways he went down were unique to him, in a similar manner to the case of Georg Simmel a few intellectual generations earlier (Grimshaw, 1983). This leads some to claim that Goffmanian studies are much less of an institutionalized grouping or movement, and are instead a loose configuration of scholarly dispositions, bound together by a certain sort of mentality, involving special sorts of sensitivity to certain kinds of interactional dynamics (Jacobsen, 2009, p. 4).

Goffman's apparent idiosyncrasy also leads to a further crack in the otherwise positive framings of him prevalent today. The uniqueness of his intellectual vision is seen to be generally rooted in, if not in fact directly created by, the oddities of his personal character (Winkin, 1999; Shalin, 2013). The latter is widely asserted to encompass various traits: elusiveness and (a possibly highly cultivated sense of) being (deliberately) enigmatic (Lemert, 2003); a tendency to avoid referring to other authors, thus making his texts and the ideas in them seem more innovative and less reliant on previous thinkers than they actually are (Collins, 1986); and characterological dispositions toward mocking others when not ignoring them, as well as coldness, superciliousness, and disdain (Delaney, 2014, p. 89).

Explanation of such matters by exegetes oscillates between two poles. First, many authors like to paint Goffman in language derived from his own works. Thus, he is seen to be the masterful crafter and wearer of multiple masks, each put on for different audiences. The masks both create and express multiple different Goffmans in the plural. The proliferating personas of “Goffman” are such that the entire set of masks is irreducible to any supposed personal essence (Fontana, 1980; Delaney, 2014).

That thought is at odds with the essentializing accounts of Goffman's personality offered by other exegetes. The idiosyncrasies of Goffman's intellectual practices are taken by them to be rooted in the oddities of Goffman's character. This was in turn shaped by the social-psychological circumstances of twentieth century North America (Shalin, 2013). He developed the kind of vision he did because he was an “outsider” in various ways: a Canadian in the US, a Jew in gentile society, a short man in a world of mostly taller men, an intellectual in (what he may sometimes have perceived as) a philistine academic world, and so on (Delaney, 2014).

Out of these circumstances are said to have come all the following Goffmans. Thus there is: Goffman the provocateur, both in academic debate and in everyday interactions, “a student of civility whose standards he flouted” (Shalin, cited at Deegan, 2014, p. 72); Goffman the joker and jester, whose fun-making could turn deadly serious in an instant (Dawe, 1973, p. 248); Goffman the competitive individualist who was always keen to self-aggrandize and put down others (Delaney, 2014, p. 89); Goffman the detached cynic, appraising human foibles from Olympian heights, vs. Goffman the passionate and engaged social critic (Strong, 1988, p. 230); Goffman the social conservative (Gouldner, 1970, p. 378), vs. Goffman the social radical and champion of the underdog (Posner, 2000; Cavan, 2014, p. 65); Goffman the overly eclectic and inconsistent magpie, who changed conceptual tack with every major new study (Strong, 2013) vs. Goffman the intellectual systematizer, whose whole intellectual career, some interpreters allege, centered on a very few basic empirical and theoretical concerns, dealing with interaction and modes of civility (Williams, 1986, p. 349), such as performances (Chriss, 1996), and selves, interactional encounters, and frames (Treviño, 2003, p. 25).

Proliferating personae and multiple masks seem the obvious tropes to describe this protean and eminently elusive character, a figure whose status has continued to grow ever greater over the last four decades. There is a widespread notion today that it is precisely the polyvalent nature of Goffman and his work which makes him an intellectual great, worthy of continuing attention, and a guide for future analyses of the kinds of phenomena he himself is taken to have initiated. Posner (2000, p. 99–100) argues that, while in his writing Goffman “does not ‘give' many messages about himself, he clearly ‘gives them off”'.

It is the seemingly endless interpretability of his work which in part stimulates later analysts to return to his writing again and again. The apparently great semiotic or hermeneutic openness of Goffman's texts may also encourage or license other scholars to define him in ways that suit their pre-existing categorisations, and to appropriate his ideas in ways that suit their own purposes (Wexler, 1984, p. 41–2).

The later Goffman warned explicitly of this problem, and advocated instead another way of reading any author:

It seems to me that you can't get a picture of anyone's work by asking what they do, or by reading explicit statements in their text what they do. Because that's by and large all doctrine and ideology. You have to get it by doing a literary kind of analysis of the corpus of their work (Goffman, cited in Verhoeven, 1993, p. 313).

Constructions of Goffman's legacy involve scholars of different types both “extending, refining, or at least applying Goffman's ideas,” while possibly at the same time compromising them by tailoring them “to fit their own professional (and other) convictions and commitments” (Rogers, 2003, p. 67). Smith (2006, p. 399) worries that “one of the perversities of Goffman interpretation has been for commentators to fail to ponder the detail of his writings, with the result that his often qualified and conditional statements get reduced to simplistic pictures,” as seen in simplified classroom teaching which reduces a life's work to a few ideas about theatrical performances of self.

While some see the possibility of Goffman's work meaning “just about anything to anyone” as a bad thing (Hillyard, 2010), and while others plot out how his ideas have been appropriated by many different sorts of actors, each changing and adapting them in turn (Morawski, 2014), still others again regard the polysemy of his writings as an ongoing opportunity to take his ideas in novel directions in new contexts (e.g., Scheff, 2006; Jacobsen, 2009; Persson, 2019).

## Classifying Goffman

Scholars both before and after his death have often sought to position Goffman as a certain type of scholar. Thus, beyond any simple identification of him as a “sociologist,” he has been understood as variously a phenomenologist (Vester, 1989; Smith, 2006), anthropologist, social psychologist, ethologist, linguist (Strong, 2013), semiotician (MacCannell, 1983), existentialist (MacCannell, 1983), deconstructionist (Clough, 1990), and interdisciplinary analyst of social power (Rogers, 1977; Jenkins, 2008).

One can also seek to classify Goffman in terms of identifying stages of his intellectual career. Winkin and Leeds-Hurwitz (2013, p. 59) point to three—roughly chronological—Goffmans: ‘the one who describes the world as “cynically manipulative,” the one who sides with the underdog, and the more technical Goffman' of the later work on frames, language, and conversation.

The apparently multiplicitous nature of “Goffman” is also testified to by the plethora of concepts, classifications, and taxonomic systems he created throughout his working life, with Williams (1988, p. 88) reckoning the total at nearly 1,000 classificatory terms. Collins (1981b, p. 222) summarizes what he takes to be the main ones:

Face-work, deference and demeanor, impression management, and the presentation of self; frontstage and backstage, teams and team-work, discrepant roles; a typology of secrets: dark, strategic, inside, entrusted, and free; moral careers, total institutions, and ways of making out in them; commitment, attachment, embracement, engagement, and role distance; focused and unfocused interaction, face engagements, accessible engagements, situational proprieties and improprieties, and the tightness and looseness of situation rules; vehicular units and participation units; territories of the self; personal space, use space, turns, information and conversational preserves; territorial violations; markers and tie-signs; supportive interchanges (access rituals) and remedial interchanges (accounts, apologies, body gloss); frames, keyings, fabrications, frame-breaking and out-of-frame activity.

The very multiplicity of classifications can be taken as evidence that Goffman was *not* a “theorist,” social or otherwise, but instead a taxonomist of social behaviors. As Collins (1988, p. 43) puts it, much of Goffman's work “looks like a microsociological Linnaeus, laying out classifications and modestly waiting for some later Darwin to bring these materials into an explanatory theory.”

The recognition of the multiple intellectual faces of Goffman involves the interplay of (at least) two key elements. First, different interpreters have selectively highlighted certain conceptual aspects of his work, while downplaying or ignoring others (Morawski, 2014). Second, the works themselves contain multiple intellectual influences. Some of these are more apparent, some less so (Collins, 1986). There are intellectual currents at play in his works that he may have referred to obliquely or not at all. His writing style was such that exegetes can discern these different currents, and then they may either identify the criss-crossing of those currents, or alternatively they may identify some currents as the dominant ones, or even as the keys to understanding what is taken as the “essential” nature and meaning of many, or most, or even all, of his writings.

In the voluminous literature on Goffman, both before and after his death, one can identify three ideal-typical forms of interpretation of his work:

1) **“Essentialist” interpretations:** The work is fundamentally a version of one specific kind of theory, with some other less important and supplementary elements added to it.
2) **“Confluence” interpretations:** The work is best understood as a unique intermingling of multiple theoretical currents (including some of those listed above), but irreducible to any or all of them.
3) **“Systematizing” interpretations:** The work constitutes a *sui generis* totality, and throughout all of it, key themes and concepts are at play. The latter are then reconstructed systematically by exegetes pushing this sort of interpretation.

In *essentialist interpretations*, the whole body of work is treated as a (relatively) unified whole, which can be relatively easily located within widely recognized schools of thought, in sociology or elsewhere (Jacobsen and Kristiansen, 2015). The risk here involves caricaturing what may better be viewed as a more complex set of different but related intellectual projects, which Goffman pursued at different points in time (Williams, 1986, p. 348; Smith, 1989, p. 20).

Within sociology, Goffman has often been described as “essentially” various sorts of things. For some he is basically a follower, or the late twentieth century instantiation, of Georg Simmel, regarding his eye for telling details, and his acute sense of the morphology of social interaction (Grimshaw, 1983, p. 148; Smith, 1989). For others, he is essentially a Radcliffe-Brown sort of structural anthropologist (Denzin, 2002). For others again, he is essentially a micro-functionalist, a Talcott Parsons of small-scale interactions (Brown, 1977).

Probably the most ubiquitous essentialist reading of Goffman involves claiming he was ultimately a Symbolic Interactionist. This is the predominant representation of him in textbooks (Brown, 2003; Carrothers and Benson, 2003). But this is an interpretation which Goffman explicitly distanced himself from, indicating that Symbolic Interactionism was too vague on how interactions were concretely organized (Verhoeven, 1993, p. 334–5). For Rawls (1987, p. 145), it is Goffman's (1983) proposal of the notion of an interaction order *sui generis* which distinguishes him from most brands of interactionist sociology, as the latter do not consider interaction as a domain *per se*, but instead study how interactions either reproduce or create social institutions.

Moreover, Symbolic Interactionism has various constituent features, and to label Goffman in that general way does not indicate which of these features he was most influenced by. One element is the social behaviorism of George Herbert Mead, where visible conducts are examined rather than internal states of mind. Goffman (1967, p. 3) could be described as adopting such a behaviorist approach in at least some of his work, such as *Interaction Ritual*, which begins by identifying—in the gendered parlance of the time—the object of study not as “*men* and *their moments*,” but rather as “*moments and their men*.”

Goffman has also been portrayed as essentially a social structuralist (Gonos, 1977; Denzin and Keller, 1981). This interpretation appeared as a reaction to the Symbolic Interactionist one. As Smith (1999, p. 4) notes, “structuralist readings applaud Goffman's sociology for downgrading individual agency by insisting on the determinative role of occasions, frames, and associated semiotic codes.” He adds that “this is useful to a degree but unfortunately neglects Goffman's compensatory awareness of interactants' capacity to improvise creatively when faced with insults, duress, frame ambiguities, and the like.”

A more potentially subtle form of structuralism comes into view when interpreters understand Goffman as fundamentally a Durkheimian or neo-Durkheimian (Collins, 1975, 1981a,b, 1988; Miller, 1982; Cheal, 1988; Cahill, 1994; Hacking, 2004). Besides his interest in the form-giving capacities of norm-driven and norm-enforcing social rituals, the sacred qualities of the individual in general, and the face in particular, in modem societies, is also a theme apparently running like a golden thread through much of Goffman's output (Collins, 1988). On this view, both Durkheim and Goffman are oriented to the classic Hobbesian question of how social order is possible, including within apparently highly individualistic modern societies (Berger, 1973, p. 356; Collins, 1980, p. 173).

The difference between Durkheim and Goffman here, according to Burns (1992), is that the former's notion of “society” stresses its robustness and durability, while the latter's “interaction order” is in some ways fragile, and in constant need of repair work by participants. For Rawls (1987), Goffman both extends, and in some ways goes well beyond, Durkheim's thinking. The latter's focus on how individuals are subject to institutional constraints on their thoughts and actions is too limited. Goffman has shown how there are other sorts of social constraints which account for much social action. It is the interaction order itself which accounts for the nature and operation of many social constraints. Goffman's conceptual innovation here means that his work can be understood as “adding the second volume to what Durkheim began” (Rawls, 1987, p. 145).

Many commentators have turned away from essentialist interpretations of Goffman, agreeing with Atkinson (1989, p. 59) that Goffman “defied categorization in relation to a[ny] particular school or tradition of sociological theory.” Turning to *confluence interpretations* of Goffman, which understand his work as a unique meeting point of various theoretical orientations, the specific confluence of theoretical elements that an author identifies can be assessed by them as more or less internally coherent. Both the combination of the elements and their relative coherence may be seen to mutate over time, as Goffman moved from one project to another. This sort of interpretation often takes a more historical orientation than does the identification of supposed essences, rooting Goffman in the broader intellectual currents of his times.

Thus Morawski (2014, p. 299) understands the work as coming out of the 1950s cultural world characterized by “phenomenology, existentialism, psychoanalysis, social criticism, avant-garde literature, and expressionist art.” Relatedly, Manning (2016b, p. 88) stresses the voracious reading that Goffman engaged in, both early in his career and throughout it:

By the time he completed his dissertation in 1953, he was steeped in the writings of the Chicago School, the sociology of Max Weber and the German methodological debates that framed his work and that of Simmel and others, the development of Emile Durkheim's sociology, leading to the breakthroughs in *The Elementary Forms of Religious Life*, Freud's work and the development of psychoanalysis generally, the existentialism of Sartre and Camus and of course the full complement of writings concerning Parsons' voluntaristic theory of action and the reactions to it by Merton and others. In addition, Goffman had a tremendous knowledge of studies of animal behavior and a dazzling array of other subject areas.

Understanding Goffman's work as a confluence of multiple theoretical influences need not stop an interpreter seeking to identify the most important influences. Thus Manning (2016a, p. 145) selects from all the many sources Goffman was exposed to three vital ones: “the great triumvirate for Goffman was Durkheim, Freud, and Parsons. Goffman is in the lineage of Durkheim, he absorbed and then reacted against Freud, and he is a version of Parsons.”

But other interpreters can also identify what are for them alternative crucial influences on Goffman, making up the unique intellectual constellation that was or is “Goffman.” One could, for example, argue that Goffman's exposure to J. L. Austin's ordinary language philosophy was just as important as those sources Manning points to above (Duranti, 2009). Alternatively, Jacobsen (2009, p. 18) argues that Goffman “can be described as a Gordian knot made up primarily by Simmelian, Durkheimian and Sartrean sources of inspiration, which he mixed into his own strong cocktail and which with varying impact permeated different stages and parts of his work.”

*Systematizing interpretations* of Goffman seek to reconstruct what they take to be the essential intellectual properties that run throughout his *oeuvre*. These imply that Goffman was indeed a “systematic social theorist,” as Giddens (1988, p. 255) framed matters, but that the systematicity must be identified by others, and bolstered by them where necessary. According to Rawls' (1987, p. 136) systematizing interpretation, Goffman's sociology is rooted in “the idea of an interaction order sui generis which derives its order from constraints imposed by the needs of a presentational self rather than by social structure.” These constraints “not only define the interaction order, but also may resist and defy social structure.” Therefore “the fact that persons must commit themselves to the ground rules of interaction in order for selves to be maintained is treated by Goffman as a moral, not a structural imperative” (Rawls, 1987). Thus, Goffman's intellectual system, as reconstructed by Rawls, is nothing less than a radical re-grounding of sociology in the direction of the interaction order, going beyond older ideas of social structure, while retaining a sense of “loose couplings” between interaction and social structures (Goffman, 1983).

Meanwhile, the systematizing interpretation offered by Manning (1992) has distilled from Goffman's writings four broad thematic areas, which Manning claims coalesce into an overarching schema labeled as SIAC. The schema comprises a “general theory” of face-to-face interaction. The acronym stands for *situational propriety* (whereby the meanings of social action can be made sense of only with respect to the situation within which actions are played out); *appropriate involvement* (the human capacity to give or withhold attention to whatever activity is at hand, involving rules of entry to or exit from participation in social encounters, and signaling to others if one is a ratified participant in them or not); *accessibility* (rules and rituals guiding appropriate levels of involvement in different types of gatherings); and *civil inattention* [means by which people acknowledge others' presence while maintaining acceptable levels of social distance (see also Manning, 1989, 1991, 1993, 2020)].

While Manning's presentation is more an exercise in creating a systematic framework for micro-sociology of interactions, and Rawls' account is more an exercise in the philosophy of social science, nonetheless all such endeavors to systematize Goffman have been criticized for missing out those things—particular concepts, specific studies, or that most elusive thing, the general “spirit” of Goffman's writing—which the critics feel have unfortunately been marginalized in, or left out of, the proposed systematization (Hartland, 1994, p. 252).

## Using Goffman for the purposes of social theory

Was Goffman some sort of “social theorist,” and are his works contributions to something called “social theory”? This is how Giddens (1988) has framed issues of Goffman's intellectual nature and significance for social science, and his framing has been highly influential in discussions of Goffman over the last several decades.

Goffman and his works certainly can be, and have been, treated as such. Many of those self-identifying as “social theorists,” including various eminent ones, have mined his texts for materials they can use in their own theoretical constructions (Fine and Manning, 2003, p. 56). Indeed, one might say that Goffman has become over the last four decades a customary, perhaps even obligatory, reference point for social theorists wishing either to deal with what are taken as micro-sociological issues, or to find conceptual means to connect what are assumed to be micro-level phenomena to macro-level ones (Chriss, 1995).

This is a point attested by no less an authority than Bourdieu (1983), whose own theoretical constructions in fact drew relatively little upon Goffman, at least explicitly or apparently. The appropriations of Goffman's writings in this regard range on a scale from theoretical constructions minimally informed by Goffman to those bearing a major debt to him (for an overview, see Jacobsen and Kristiansen, 2015). Yet appropriating Goffman for one's own theoretical purposes may be more challenging than it at first looks (Hartland, 1994, p. 251).

A more minimalist use of Goffman is by Luhmann (1979), who derives his understanding of inter-personal trust from Goffman. A more substantial drawing on Goffman is carried out by Giddens (1984) himself, in the construction of his version of structuration theory. Giddens takes from Goffman cues for better understanding practical consciousness and everyday routines, understood as building-blocks necessary in a general social theory that can successfully connect psychological and structural phenomena (Hartland, 1994, p. 252). “The routinization of encounters is of major significance in binding the fleeting encounter to social reproduction and thus to the seeming ‘fixity' of institutions” (Giddens, 1984, p. 72).

Giddens reworks Goffman as a theorist, or at least proto- or quasi-theorist, of social reproduction. Simultaneously, Goffman and ethnomethodology are drawn upon by Giddens to show that everyday routines are not mechanically enacted, but rather are subjected by participants to constant monitoring and adaptation (Jacobsen and Kristiansen, 2015). Rawls (1987, p. 136), however, takes Giddens to task for turning Goffman into a theorist of social reproduction, when she contends that he is in fact a theorist of the dynamics of the interaction order, which is analytically and, perhaps, empirically a separate domain from social systems and their reproduction dynamics.

While Giddens uses Goffman in a significant but still limited manner, the very name that Collins (2004) gives to his own general theory, involving the analysis of “interaction ritual chains,” illustrates the explicit and large indebtedness to Goffman in the construction of the theory. This is a general account of “momentary encounters among human bodies charged up with emotions and consciousness because they have gone through chains of previous encounters” (Collins, 2004, p. 3). Thus “in social occasions with high levels of intersubjectivity, emotional entrainment produces emotional energy,” and in those occasions “with a high degree of ritual intensity … old social structures are torn up” and new ones come into being (cited at Jacobsen and Kristiansen, 2015, p. 65).

In other words, Goffman can be appropriated to theorize social change as well as social reproduction. An alternative use of Goffman to understand how the latter works, in terms of how cultural orders impose constraints on interactions, is proposed by Turner (2002). Another possible use of Goffman is offered by Hancock and Garner (2011, p. 320), who read him as a general theorist of human life, which is understood as “a constant agonistic pull between absolute stability and absolute instability.”

Goffman has also been a source of inspiration for feminist sociological theory (Deegan, 2014). Taking off from Goffman's points about symmetrical and asymmetrical relations between participants in interaction, including in conversations, feminist work (e.g., Fishman, 1978) has conceptualized, for example, how and why women do more work than men to generate ongoing flows of communication. West (1996, p. 360–1) summarizes the feminist use of Goffman as involving: appreciation of how gendered social power works in spoken and other interactions; the identification of mundane conversations as a site of such power dynamics; allowing for development of the insight that “the exercise of power is perhaps most effective when it is muted, if not euphemized”; and pinpointing—independently of, and before, Butler's (1990) influential contributions—the performativity of gender categories, whereby mundane processes like labeling toilets as “male” and “female” are presented as consequences of biological, sex-based difference, but in fact the labeling practices create and reproduce such distinctions.

As to the uses made of Goffman's notions by ethnomethodology and Conversation Analysis (CA), both of these are in some ways far from “mainstream” social theory, at least as far as polemics for and against them are concerned, where both fields have often been rhetorically constructed as being in some way or another “against theory” as conventionally understood and institutionalized. The relations between Goffman and ethnomethodology are complex. Goffman explicitly distanced himself from what he saw as ethnomethodology's extreme “subjectivism” (Goffman in Verhoeven, 1993, p. 327). As developed by Sacks (1984) and others, CA took up the idea that talk-in-interaction is a fundamental domain which can be studied in its own right, by focusing on the procedural basis of conversation's production. “This basis was conceived as a site of massive order and regularity, whose normative organization and empirical regularities could be addressed using the sorts of basic observational techniques that a naturalist might use in studying animals or plants” (Heritage, 2009, p. 302–3). Thus, the apparently a- or anti-theoretical nature of CA was in fact based to a significant extent on a general—“theoretical”—contention formulated by Goffman.

## Goffman as a “social theorist”?

Goffman has certainly been treated as a fellow social theorist by many of those who are engaged in such kinds of knowledge production (Giddens, 1988). But there is no consensus in writings about Goffman as to whether he was or is a “theorist,” and whether his works are indeed “theoretical” *per se*, or instead are just quarries of useful information and scattered ideas useful for those who would do theoretical work (Deterling, 2009).

The case for treating Goffman as a “social theorist” has been made in various ways. His was an “acute and far-ranging theoretical mind,” as his long-term associate Dennis Wrong (1990, p. 9) put it, and he was highly versed in American, French, German and British social theory from an early point in his career. He can be presented as, at root, a major, if not *the* most important, “sociological theorist of everyday life” (Brewster and Bell, 2010, p. 46).

One could follow Manning (2000, p. 283) in arguing that “despite the many illustrations, telling examples, and subtle observations, Goffman was an abstract thinker who sought to expose the most general characteristics of face-to-face interaction … [T]he analysis of face-to-face interaction is as much an abstraction as the ‘behavior' of corporations or New York crime statistics … [while] even his ethnographic studies were abstract and self-consciously theoretical.” On that view, *Asylums* (Goffman, 1961) is not a case study of a specific empirical entity, it is an ethnographic analysis of *a concept*, the total institution. Manning (2000, p. 283) goes on to make the case for Goffman as a theorist of both trust and deception in interaction.

Moreover, Goffman's (1974, p. 5) analogy in *Frame Analysis* can be read as an indication of his interest in creating general theory:

A game such as chess generates a habitable universe for those who can follow it, a plane of being, a cast of characters with a seemingly unlimited number of different situations and acts through which to realize their natures and destinies. Yet much of this is reducible to a small set of interdependent rules and practices. In other words, beginning with a few very simple rules governing how each of the six pieces may move, chess is the sort of game that nevertheless provides a nearly infinite variety of possible endgames and conclusions. If the meaningfulness of everyday activity is similarly dependent on a closed, finite set of rules, then explication of them would give one a powerful means of analyzing social life.

For Chriss (1993), Goffman went out into the world and observed the enormous variety of human activity, hoping one day to discover a general theory of interaction.

Such accounts present Goffman's theoretical capabilities and orientations as advantages. Conversely, a more minority position stresses the disadvantages: Goffman was in fact too general, abstract, and “theoretical” a thinker. For Strong (2013, p. 234–235), Goffman was not an empirical researcher “in any conventional sense. He was a theorist working in an unexplored area, trying to make some sense, as best he could, of a huge and unfamiliar terrain.” There is consistency in theoretical ideas throughout his work. Thus he was not like those “systematic explorers who plod after him” (Strong, 1988, p. 230), persons who do detailed empirical work that may yet yield surprising findings not in line with Goffman's theoretical assumptions.

The implication here is that Goffman was too little attuned to empirical occurrences because of the marked theoretical assumptions, derived from Durkheim and other sources described earlier, which he consistently carried with him, regardless of which subject matter he was working on. According to Dawe (1973, p. 247), Goffman “rarely misse[d] an opportunity to draw large-scale conclusions from his small-scale observations, however incidentally he may [have] present[ed] them. For example, the careful analysis of remedial interchanges in *Relations in Public* (Goffman, 1971) is followed by an unconvincing attempt to link the manners, styles, and rituals thus revealed to ‘core moral traditions of Western culture' ”. This was an inappropriate Durkheimian conjoining of the micro and the—possibly spuriously conceived—macro.

Denials of Goffman's status as “social theorist” can take various forms. In a much-reproduced statement from his posthumous tribute to Goffman, Freidson (1983, p. 359) concluded that “Goffman's work has no systematic relationship to abstract academic theory and provides no encouragement to attempts to advance such theory. What gives Goffman's work a value that will endure far longer than most sociology is its intense individual humanity and its style”—another iteration of claims as to Goffman's “inimitability.”

One can also focus on Goffman's alleged intentions to say that he never attempted to be a “theorist,” or to produce “theory” in any conventional sense of that term (Scheff, 2003, p. 59). For Fine and Manning (2003, p. 34), throughout his whole career Goffman “did not attempt to develop an overarching theory of society; nor did he raise issues that speak to transhistorical concerns of social order.” Strong's (2013, p. 147) sardonic take on that point is that “Goffman systematically refused to take part in the inter-galactic paradigm-mongering which conventionally passes for really serious sociology,” instead focusing on detailed analysis of the interaction order.

Another way of denying Goffman's position as a “social theorist” is to claim that what “Goffman left us is not so much a theory, as a formal analysis in the manner of Simmel” (Treviño, 2003, p. 26). This preserves Goffman's status as a great thinker, akin to Simmel, but denies that he was producing “theory” in the sense of general accounts of why things happen as they do. Instead, “his near-obsession with the classification of social behavior—that is, with its *organization* and *description—*did not allow him to develop a proper *explanation* of it … [C]ausal analysis always took a backseat to analytical arrangement … [E]ven when he is at his conceptual best—when he renders rich and detailed depictions of social life through the use of countless lively illustrations—Goffman's examples are not primarily intended as exemplifications of social behavior; instead their first goal is to exemplify the usefulness of a *classification* of social behavior” (Treviño, 2003, p. 26).

Thus, while the typologizing Goffman “brought serious conceptual order to an understanding of the micro-social world,” this was at the level more of taxonomy than of “theorizing” *per se* (Treviño, 2003, p. 31).

Alternatively, some commentators present Goffman as existing outside of conventional academic roles of theorist, ethnographer, empirical researcher, and so on. Scheff (2003, p. 51) sees Goffman as doing something much more fundamental than any of those: “Goffman's work does not make much, if any, contribution to theory, method, or empirical evidence as these categories have come to be understood in social science. It is conceivable, however, that we might be dealing with something more primitive, preliminary to theory, method, and evidence.”

Fine et al. (2000, p. ix) present Goffman as going beyond conventional academic roles and classifications in another way: “he was neither a traditional ethnographer nor an orthodox social theorist: his ethnography was too theoretical and his theory too ethnographically rich.” In other words, Goffman is just too protean a figure to be reduced to the mere status of “social theorist.” While for some he was not intellectually systematic enough to reach this status, for others he was markedly beyond it.

## Goffman between theorizing and methodology

Giddens (1988) posed the question of whether or not Goffman was a “social theorist,” thereby setting up issues of Goffman's sociological and social scientific importance and influence in terms of a defined role and intellectual type, a theorist of the social realm. We contend it is more interesting and productive to consider the nature of his “theorizing,” understood as an active and creative act which juxtaposes empirical data and theoretical concepts in multiple emergent constellations expressed in writing.

Swedberg (2016) proposes to analyse what different sorts of scholars do when they “theorize.” Applying this perspective to Goffman would involve dropping the possibly overly static terms “theorist” and “theory,” the former understood as the fabricator of a finished end-product, and instead look at the nature of Goffman's practices as an active “theorizer,” examining his characteristic processes of creating concepts and claims, rather than focusing, as many exegetes tend to, on the end results of those processes, namely his published works. How then to present Goffman's “theorizing” practice?

Delaney (2014, p. 96) likens Goffman to Wittgenstein in that both were masterful at *ekphrasis*, “vividly graphic verbal representations that seem to capture the quiddity of whatever is being described in a compelling synthesis of form and content.” On this account, Goffman's theorizing has two major components. In a manner that most theoreticians do, Goffman used illustrative examples to render conceptual points concretely, succinctly, and vividly. But less conventionally, Goffman also engaged in theorizing in ways akin to Hegel's use of “concrete universals”:

Examples had for him at once their own integral validity as data unto themselves in addition to a wider, representational, logico-formal force as illuminating indicia of more general features of social life. Once formulated as a discrete data-unit—and Goffman was most emphatic on the crucial importance of devising good units—they can serve as generative models or microcosmic epitomes of abstractable conceptual complexes to be compared and correlated with other data-sets (both like and unlike), systematically played with in an elastically topological manner, then eventually put together like a jigsaw puzzle, as he was fond of saying (Delaney, 2014, p. 96).

We may also expect Goffman's classroom teaching methods to illustrate his means of engaging in on-the-spot theorizing. Delaney (2014, p. 97) points out that Goffman repeatedly informed students that “thought-provoking data are lying all around one for the taking, if only one had wit enough, and the attention span, to look.”

Like Sherlock Holmes castigating the obtuse Dr. Watson, one must not just *see*, but also *observe*. Effective observation meant being attendant to the fact that a lot of “vernacular data comes complete with its built-in frame-structure sticking out, virtually begging to be explicated, then formally correlated with other datasets, or otherwise put to good use” (Delaney, 2014).

Another way of representing Goffman's theorizing is to say that it involved developing, honing, and deploying conceptual and linguistic “weapons by which to assail the fictional facades that constitute the assumptive reality of conventional society” (Manning, 1980, p. 263). Scheff (2003, p. 55) argues that Goffman's theorizing proceeds by “engineer[ing] a continuing clash between the taken-for-granted assumptions in our society and his incongruous metaphors and propositions.” This involved constantly coining phrases that are “accurately improper.” That is, they describe what is essentially going on in specific sorts of encounters, in ways that make sense to a reader precisely because they import into the understanding of the given scenario ideas and images not usually associated with it in their mental and cultural landscape. A certain shock-value attaches to these images and tropes: the reader sees the scenario in a new light, because it is connected to something that conventional thought does not usually connect it with. The connection presents “an enlightening glimpse of … another reality behind the conventional one” (Scheff, 2003, p. 55). For example, we are led to see that a conman and a psychiatrist are doing much the same sorts of things when they do what they typically do to other people.

Dellwing (2016, p. 126) argues that “Goffman was opposed to science that just reproduces and orders concepts, and [also was] opposed to his students using his concepts as easy tools. Instead, he recommended they train their own horses.” If we accept that claim, then a focus on his modes and means of theorizing, rather than on constructing a finished “theory” or set of axiomatic theoretical principles through exegesis of his writings, possibly seems closer to what he apparently intended his work to achieve, although what exactly that was is always open to contestation (Smith, 2006, p. 1).

Goffman's general intellectual practice, including his “theorizing,” was inexorably bound up with methodological issues of data collection and analysis. Rawls (2022) notices a tendency among critics of Goffman to express wonder and confusion as to the scientific status of descriptions of interaction that run through his works. Can vignettes from novels or newspaper articles, for example, really count as sound data that give accurate descriptions of specific sorts of interaction?

A medley of scholarly voices has taken aim at the perceived inadequacies of Goffman's methodology. Running through the voluminous secondary literature is the theme that while Goffman may have been a brilliant thinker and sometimes a brilliant observer, he was a less than impressive methodologist. Sharrock (1999, p. 119) sums up the views of many others when he alleges that Goffman's weakest area was general methodology. Bourdieu (1983) even went so far as to claim that Goffman did not create a worked-out methodology at all.

The apparent strength of Goffman's *modus operandi*—that it seems to be exceptionally open-minded in its drawing upon any and every kind of data source that Goffman could usefully lay his hands on—is condemnable both as an overly loose eclecticism, and as unfit to capture the real intricacies of the Interaction Order. As Schegloff (1988, p. 132) puts it: “Goffman may well have shown us how far you can go with real-time observation, clippings and vignettes. With them he helped show the direction in which what must surely be a central domain (i.e., the Interaction Order) for the social sciences could be found, but something different may be required to actually find it.”

From that sort of point of view, Goffman's tendency to paraphrase, or even invent, lines of talk is as unacceptable as his penchant for bending data to fit pre-existing forms of argument. If “Goffman based his observation on memory and prodigious intuition,” while Harvey Sacks and other pioneers of Conversation Analysis “began to capture interactional details by recording them on tape,” it is the latter who seems to have created a methodological pathway into the future, while Goffman has created a dead-end (Pallotti, 2007, p. 3).

Such critique is again tied up with with the problems of the apparent irreproducibility and inimitability of his work, and the alleged quirkiness of the man himself (Fine and Manning, 2003; Delaney, 2014). The idiosyncrasy applies variously to these matters: his capacities to grasp things other people coming before him could not grasp; to his distinctive research practice—gathering data from every possible source, hither and thither, and then subjecting them to his own unique interpretative abilities; and to his writing style—brilliant but non-reproducible (Daniels, 1983). To carry out Goffman-style analysis it seems one would have to be Goffman himself, for his approach to data gathering, and what he then does with the data, is so idiosyncratic to the man himself (Anderson et al., 1985).

The critiques of Goffman as methodologically loose to the point of incoherence generally are formulated by scholars whose own methodological predispositions are clear (at least to themselves), and who are convinced of the superiority of their approach to Goffman's apparently disorganized eclecticism. To that extent the critiques are tendentious: they assume the superiority of one method over another. The implicit message often seems to be thus: to study the Interaction Order properly, you must do it our way, and any other ways will be less effective or simply misguided. But the problem of narrowness of vision then becomes apparent. As Strong (2013, p. 149) pointed out:

Naturalistic data comes in all shapes and sizes. Most of its analysts, however, have extraordinarily narrow preferences. Some cluster round tape-recordings, others around pictures and yet others around the printed word; some, notebook in hand, spy on lives or institutions, others will only watch video; some swear solely by interview, others believe in nothing but observation.

Yet the spirit of Goffman's entire oeuvre arguably seems to have been to collect any kind of data, in any manner, that might provide material that may at some later date prove useful in some way. Such an approach strongly resonates with Feyerabend (1975) position in the philosophy of science that any sort of data collection method is acceptable if it allows for creative thinking in the process of the handling of data. As Becker (2003, p. 660) argues, Goffman for example “felt very strongly that you could not elaborate any useful rules of procedure for doing field research and that, if you attempted to do that, people would misinterpret what you had written, do it (whatever it was) wrong and then blame you for the resulting mess.”

Moreover, Goffman (1981a,b) was critical of attempts by its proponents of one methodology seeking to monopolize the study of the Interaction Order, be that the transcript-driven approach of Conversation Analysis or any other form of data collection and analysis (Ranci, 2021). Strong's (2013, p. 150) gloss on these admonitions is for the student of the Interaction Order not to “get uptight about a single data-source,” because for Goffman, “there was no point in analyzing a single data-source. What is for the most of us the end of our enquiry was for Goffman merely the beginning. Having done his ethnography or collected and analyzed his initial data, he would then scour a host of other more readily assimilable data sources to see what light they threw upon the matter in hand.”

That point raises issues about the roles of recording technologies—from tape-recorders and video, through to photography of both longer- and shorter-distances, and physiological body monitors, and so on—in the operation of post-Goffman studies of the Interaction Order. In a recent consideration of the development since the 1970s of the broad field of micro-sociology and cognate areas, Collins (2016, np) describes a feedback loop “between innovation in research methods and theoretical concepts,” such that today the “deepening spiral between research technology and theory also improves traditional methods of observation and interviewing; it sharpens our ethnographic eye as to what to look for and what kinds of detail to probe for in our questions. Today's ethnographer can say, *I am a camera*, echoing Christopher Isherwood describing Berlin in the 1930s.”

For Collins (2016, np), the evolution of technological devices that can record interactional phenomena with great accuracy can only mean better quality data than was hitherto available, and that data will allow much better understanding of a wide range of phenomena hitherto less susceptible to investigation, such as “mechanisms of mutual awareness, the causes and consequences of a variety of shared emotions, and the patterns of rhythmic entrainment that together determine levels of solidarity and emotional energy.”

This is no doubt true in one way. But the technological optimism of Collins' account gives one pause for thought. Must the increasing reliance of analysts of the Interaction Order on the data created by such devices side-line or outlaw the more eclectic approach to data-gathering that Goffman practiced? Does the shift toward the apparently objective apparatuses of the natural scientist forbid the collating and use of the kinds of materials, such as vignettes taken from novels, that the humanistic scholar—which Goffman partly was—would want to draw upon? Does a technology-oriented field of study demand that the data-gathering and analysis activities of Goffman be framed as belonging only to the pre-scientific pre-history of the field?

Given that some such technologies cost a lot of money and are operated in teams, what then of the lone scholar who would investigate the Interaction Order on their own, and cheaply too, through the collection of the diverse sorts of stuff Goffman used to do? Must studies of the Interaction Order in the future wholly depend on the largesse of funding bodies, and the success of successful scientists in playing the lottery games of scientific funding? The answers given to those questions will of course depend on which kinds of investigator are giving the answers. If there is still room in today's academy for individualist scholars who do their own thing as regards the Interaction Order—or Interaction Orders in the plural (Rawls, 2022)—outside of the framework of large funding and big interdisciplinary teams, then the example of Goffman may still serve some useful purposes.

## A Goffmanesque perspective on history and social change

Theorizing and designing methodology in ways inspired by, but not mechanically reproducing, Goffman's ways of doing such things, may yield new directions for both “social theory” and empirical research in interaction. As an example of this possibility, one might highlight Goffman's relatively untapped potential for the study of both social change and societies of the past. “Few have addressed Goffman's implicit vision of social change—and the question remains if Goffman's largely situational microsociology is at all suitable for shedding light on the impact of some of the major social and cultural transformations” (Jacobsen and Kristiansen, 2015, p. 63).

That point can be illuminated with reference to the following issues contained in his famous Presidential Address to the American Sociological Association, a text never given to the intended audience because of terminal illness, but which is often taken as Goffman's final will and testament, intellectually speaking. Goffman (1983, p. 2) reflects briefly on how taking “environments in which two or more individuals are physically in one another's response presence” as the basic point of sociological attention, means that sociological thinking's standard contrasts—such as “between village life and city life, between domestic settings and public ones, between intimate, long-standing relations and fleeting impersonal ones”—are problematised. When such things are researched and theorized in the ways Goffman suggests or implies, they are transformed from unquestioned assumptions into open questions, as to whether there are, for example, significant differences or not between urban and rural environments, or modern and pre- and non-modern ones.

Goffman (1983, p. 2) explicitly noted that identifying the “interaction order” as a domain in its own right, “loosely coupled” to social structures and systems, “provides a means and a reason to examine diverse societies comparatively, and our own historically.” He thereby opened-up the possibility of studying how a specific form of interaction order—whether in our own time and in contexts the analyst lives within, or at other points in time and space—“comes into being historically, spreads and contracts in geographical distribution over time, and how at any one place and time particular individuals acquire” the understandings necessary to operate within a given interaction order (Goffman, 1983, p. 5).

A Goffman-inspired theorizing of the historical construction of, and changes over time in, such orders, would address such matters as the specific forms take by “avoidance and presentational rituals, respectful ceremonial distance, both physical and symbolic, and explicit attestations of alter's personhood (e.g., salutations, compliments, etc.),” among other phenomena (Colomy and Brown, 1996, p. 20). A central focus here has already been set up by Goffman (1983, p. 6) himself, namely attention to asymmetries of social power in historical interactions: “there is no doubt that categories of individual in every time and place have exhibited a disheartening capacity for overtly accepting miserable interactional arrangements.”

In effect, Goffman gestured toward a novel kind of historical sociology of interaction, which would complement, intersect with, critique, and in some ways go beyond existing approaches in historical sociology, notably that of Norbert Elias, another student of interactions and the modes of manners expressed in them. If pre-modern interaction orders could be shown to be, beyond obvious sorts of differences, *generally similar* to modern ones, the radical implication would be that conventional sociological understandings of “modernity” and its alleged uniqueness and radical difference from pre-modern social orders, would be seriously brought into question and would have to be rethought profoundly.

## Discussion and conclusion

There is a school of thought that has contended that it is impossible to train future investigators, or indeed oneself, directly to follow in Goffman's footsteps, precisely because the pathways he went down were in some ways unique to him (Grimshaw, 1983). To be inspired in one's research by Goffman is to *do* the kinds of things he did in a very general sense, but it is certainly not to do so with any of his substantive concepts or findings in mind as one does so (Dellwing, 2016, p. 126).

At least three kinds of things Goffman did, and which can be imitated by others today and in the future, involved specific orientations to data collection, data analysis, and literary presentation thereof. First, we can follow Dellwing (2016, p. 131) in arguing that Goffman refused to make and police divisions between “science and non-science, reality and fiction, and research time and non-research time.” Instead, anything that he had happened to gather and seemed usable for the purpose at hand could be used. In this way “method” is a means of allowing the world to open up to the investigator in all its plenitude, and emphatically not a weapon to “declare work ‘unscientific' when it failed to follow a specific sectarian incantation” (Dellwing, 2016, p. 131).

Second, in terms of working on the data thus gathered, we can follow Williams (1988) suggestion that Goffman's intellectual practice is akin to Baldamus' (1972, p. 295) understanding of how theorizing operates, in which one simultaneously manipulates hither and thither both the things to be explained and the emerging explanatory framework of concepts being used to understand it. The implication of such a practice is that investigation is not only about the discovery of previously unknown things, which Collins' account, noted above, of the new phenomena that will be revealed by technologically assisted investigations, implies. It is also equally about attributing new significances to things that are in some ways already known.

The third element is both part and outcome of such thought processes. By generating new metaphors through such an iterative process, new light is shed on already known phenomena. And the presentation of those new forms of significance is carried out through a deft literary presentation to audiences of those matters, whereby metaphorical representation of one's claims and findings is deployed as an aid to understanding but is at the same time not allowed to shackle that understanding by becoming reified. The usefulness of the metaphor is worked out in and through the text, but its limitations are acknowledged too. When the imagery has outlasted its usefulness, it can be dropped, just as Goffman did from one specific study to another.

If the analyst's metaphor-based attributions are to shed genuinely new light on phenomena that either common-sense already understands or which previous scientific investigations have already found, then their construction will require the kind of imaginative leaps and lateral thinking that Goffman was often so good at. That imaginative element is an inalienable part of Goffmanesque investigation. Specific facts of interaction, as recorded on audio or video technology, do not speak for themselves. Instead, they must be handled by the investigator's imaginative capacities, as operationalised through her verbal dexterity, thereby coaxing them into the light of conceptual significance. The analyst must look at and listen to data in a manner highly informed by literary sensibilities, deploying and reworking metaphor-driven description and re-description as she goes.

This is neither easy to do, nor easy to teach people to do. Dellwing (2016, p. 138) is correct to say that it is “excruciatingly hard to be creative” in a Goffmanesque manner, for it requires one “to produce insight and conceptual categorization that emerge from open material and metaphor without the crutches of clear method or prepackaged systematic theory.” Conversely, while sophisticated methods and technologies of data creation, collection, and analysis may make it easier for large and wellfunded teams to do the kinds of work on the Interaction Order expected of them by their peers, there is still both room and necessity for *thinking* like Goffman, even if that means not reproducing the contents of his thought.

That means in turn that highly literate intellectuals—not quite the same thing as laboratory-based scientists or other sorts of academics—are still needed to push forward the study of the Interaction Order. At the very least, someone will still have to write the “results” up with a certain modicum of literary flair and metaphorical perspicacity, which is perhaps not the usual intellectual trade of many laboratory scientists.

The very production of analytical and conceptual significance, both wrung out of recorded data and attributed to it, is at the core of a Goffman-influenced approach to studying interaction, and so literary capacity and metaphor-mongering are capacities that must be possessed by at least some members of research teams, and not just the more senior ones. Such skills will more likely be cultivated if, like Goffman (1981b) himself admitted, team members go out of their way to develop their own literary capacities. Goffman himself owed some debt to great prose stylists, such as George Orwell, and he was unafraid in his scientific writings to cite, for example, the then-popular novels of Kingsley Amis, or to compare sixteenth century etiquette books to present-day sources concerning the conduct of human behavior (Goffman, 1971, p. 48).

Students of the Interaction Order today do not need to imitate the use of literary sources, but they could and should take a leaf out of Goffman's book(s), as it were, by being attentive to multiple sources of inspiration, in terms of how to use literary sources, and the wider analytic sensibilities they embody and afford to others, in order to wring interesting and perhaps unexpected forms of significance out of their data. If they do so, they are being Goffmanesque, or at least they are doing one, eminently productive, version of what that term may mean.

## Data availability statement

The original contributions presented in the study are included in the article/supplementary material, further inquiries can be directed to the corresponding author.

## Author contributions

All authors listed have made a substantial, direct, and intellectual contribution to the work and approved it for publication.

## References

[B1] Anderson R. J. Hughes J. A. Sharrock W. (1985). The Sociology Game. London: Longman.

[B2] Atkinson P. (1989). Goffman's poetics. Hum. Stud. 12, 59–76. 10.1007/BF00142839

[B3] Baldamus W. W. (1972). “The role of discoveries in social science,” in The Rules of the Game, ed T. Shanin (London: Tavistock), 276–302.

[B4] Becker H. S. (2003). The politics of presentation: Goffman and total institutions. Symbol. Interact. 26, 659–669. 10.1525/si.2003.26.4.659

[B5] Berger B. M. (1973). A fan letter on Erving Goffman. Dissent 20, 353–361.

[B6] Bergesen A. (1984). Reflections on Erving Goffman. Q. J. Sociol. 8, 51–54.

[B7] Bourdieu P. (1983). Erving Goffman: discoverer of the infinitely small. Theory Cult. Soc. 2, 112–113. 10.1177/0263276483002001012

[B8] Branaman A. (1997). “Goffman's social theory,” in The Goffman Reader, eds C. Lemert and A. Branaman, Oxford: Blackwell.

[B9] Brewster B. H. Bell M. M. (2010). The environmental Goffman: toward an environmental sociology of everyday life. Soc. Nat. Resour. 23, 45–57. 10.1080/08941920802653505

[B10] Brown D. (2003). Goffman's dramaturgical sociology. Teach. Sociol. 37, 288–299. 10.2307/3211326

[B11] Brown R. H. (1977). A Poetic for Sociology: Toward a Logic of Discovery for the Human Sciences, Cambridge: Cambridge University Press.

[B12] Burns T. (1992). Erving Goffman, London: Routledge.

[B13] Butler J. (1990). Gender Trouble, London: Routledge.

[B14] Cahill S. A. (1994). Following Goffman, following Durkheim into the public realm. Res. Commun. Sociol. 1, 3–17.

[B15] Carrothers R. M. Benson D. E. (2003). Symbolic interactionism in introductory textbooks. Teach. Sociol. 31, 162–181. 10.2307/3211306

[B16] Cavan S. (2014). When Erving Goffman was a boy: the formative years of a sociological giant. Symbolic Interact. 37, 41–70. 10.1002/symb.83

[B17] Cheal D. J. (1988). Relationships in time: ritual, social structure, and the life course. Stud. Symbol. Interact. 9, 83–109.

[B18] Chriss J. J. (1993). Looking back on Goffman: the excavation continues. Hum. Stud. 16, 469–483. 10.1007/BF01323029

[B19] Chriss J. J. (1995). Some thoughts on recent efforts to further systematize Goffman. Sociologic. Forum 70, 177–186. 10.1007/BF02098570

[B20] Chriss J. J. (1996). Toward an interparadigmatic dialogue on Goffman. Sociologi. Perspect. 39, 333–339. 10.2307/1389249

[B21] Clough P. T. (1990). “Reading Goffinan: toward the deconstruction of sociology,” in Beyond Goffman: Studies on Communication, Institution and Social Interaction, ed S. H. Riggins (Berlin: de Gruyter).

[B22] Collins R. (1975). Conflict Sociology. New York, NY: Academic Books.

[B23] Collins R. (1980). “Erving Goffman and the development of modern social theory,” in The View From Goffman, ed J. Ditton (London: Macmillan), 170–209.

[B24] Collins R. (1981a). On the microfoundations of macrosociology. Am. J. Sociol. 86, 984–1014. 10.1086/227351

[B25] Collins R. (1981b). “Three stages of Erving Goffman,” in Sociology Since Midcentury, New York, NY: Academic Press.

[B26] Collins R. (1986). The passing of intellectual generations: reflections on the death of Erving Goffman. Sociol. Theory 4, 106–113. 10.2307/202109

[B27] Collins R. (1988). “Theoretical continuities in Goffman's work,” in Erving Goffman—Exploring the Interaction Order, eds P. Drew and A. Wootton, Cambridge: Polity.

[B28] Collins R. (2004). Interaction Ritual Chains. Princeton: Princeton University Press.

[B29] Collins R. (2016). What Has Micro Sociology Accomplished? Available online at: http://sociological-eye.blogspot.com/2016/04/what-has-micro-sociology-accomplished.html

[B30] Colomy P. Brown J. D. (1996). Goffman and interactional citizenship. Sociologic. Perspectiv. 39, 371–381. 10.2307/1389252

[B31] Daniels A. K. (1983). Erving Manual Goffman. Footnotes, January, American Sociological Association.

[B32] Dawe A. (1973). The underworld-view of Erving Goffman. Br. J. Sociol. 24, 246–253. 10.2307/588382

[B33] Deegan M. J. (2014). Goffman on gender, sexism, and feminism: a summary of notes on a conversation with Erving Goffman and my reflections then and now. Symbolic Interact. 37, 71–86. 10.1002/symb.85

[B34] Delaney M. (2014). Goffman at Penn: star presence, teacher-mentor, profaning jester. Symbol. Interact. 37, 87–107. 10.1002/symb.88

[B35] Dellwing M. (2016). A guide to training your own horses: the flaneur approach and Erving Goffman's uninhibited research practices in sociology. Symbol. Interact. 39, 126–142. 10.1002/symb.216

[B36] Denzin N. (2002). Much ado about Goffman. Am. Sociol. 33, 105–111. 10.1007/s12108-002-1005-3

[B37] Denzin N. Keller C. (1981). Frame analysis reconsidered. Contempor. Sociol. 10, 52–60. 10.2307/2067803

[B38] Deterling S. (2009). “The game frame: systemizing a Goffmanian approach to video game theory,” in Proceedings of DiGRA 2009. Available online at: http://www.digra.org/digital-library/publications/the-game-frame-systemizing-a-goffmanian-approach-to-video-game-theory-extended-abstract/

[B39] Ditton J. (1980). The View from Goffman. London: Macmillan.

[B40] Drew P. Wootton A. (1988). Erving Goffman—Exploring the Interaction Order. Cambridge: Polity.

[B41] Duranti A. (2009). Linguistic Anthropology. Oxford: Wiley-Blackwell.

[B42] Feyerabend P. (1975). Against Method: Outline of an Anarchistic Theory of Knowledge. London: New Left Books.

[B43] Fine G. A. (2018). Review of the Goffman Lectures: philosophical and sociological essays about the writings of Erving Goffman. Contemp. Sociol. 47, 189–190. 10.1177/0094306118755396r

[B44] Fine G. A. Manning P. (2003). “Erving Goffman,” in The Blackwell Companion to Major Contemporary Social Theorists, ed G. Ritzer (Oxford: Blackwell).

[B45] Fine G. A. Manning P. Smith G. (2000). “Introduction,” in Erving Goffman, eds G. A. Fine, P Manning, and G. Smith, vol. I (London: Sage), ix-xliv.

[B46] Fishman P. (1978). Interaction: the work women do. Soc. Probl. 25, 397–406. 10.2307/800492

[B47] Fontana A. (1980). “The mask and beyond: the enigmatic sociology of Erving Goffiman,” in Introduction to the Sociologies of Everyday Life, ed J. D. Douglas (Boston: Allyn and Bacon).

[B48] Freidson E. (1983). Celebrating Erving Goffman. Contemp. Sociol. 12, 359–362.

[B49] Giddens A. (1984). The Constitution of Society, Cambridge: Polity.

[B50] Giddens A. (1988). “Goffman as a systematic social theorist,” in Erving Goffman—Exploring the Interaction Order, eds P. Drew and A. Wootton (Boston: Northeastern University Press).

[B51] Goffman E. (1961). Asylums. Harmondsworth: Penguin.

[B52] Goffman E. (1967). Interaction Ritual: Essays on Face-to-Face Behavior. New York, NY: Pantheon.

[B53] Goffman E. (1971). Relations in Public: Microstudies of the Public Order. New York, NY: Basic Books.

[B54] Goffman E. (1974). Frame Analysis: An Essay on the Organization of Experience. New York, NY: Harper and Row.

[B55] Goffman E. (1981a): Forms of Talk. Philadelphia: University of Pennsylvania Press.

[B56] Goffman E. (1981b). A reply to Denzin and Keller. Contemp. Sociol. 10, 60–68. 10.2307/2067804

[B57] Goffman E. (1983). The interaction order. Am. Sociologic. Rev. 48, 1–17. 10.2307/2095141

[B58] Gonos G. (1977). “Situation” vs. “frame”: the “interactionist” and the “structuralist” analyses of everyday life. Am. Sociol. Rev. 42, 854–867. 10.2307/2094572

[B59] Gouldner A. W. (1970). The Coming Crisis of Western Sociology. New York, NY: Basic Books.

[B60] Grimshaw A. D. (1983). Erving Goffman: a personal appreciation. Lang. Soc. 12, 147–148.

[B61] Hacking I. (2004). Between michel foucault and Erving Goffman: between discourse in the abstract and face-to-face interaction. Econ. Soc. 33, 277–302. 10.1080/0308514042000225671

[B62] Hancock B. H. Garner R. (2011). Towards a philosophy of containment: reading Goffman in the 21st century. Am. Sociol. 42, 316–340. 10.1007/s12108-011-9132-3

[B63] Hartland N. G. (1994). Goffman's attitude and social analysis. Hum. Stud. 17, 251–266. 10.1007/BF01323604

[B64] Heritage J. (2009). “Conversation analysis as social theory,” in The New Blackwell Companion to Social Theory, ed B. S. Turner (Oxford: Blackwell), 300–320.

[B65] Hillyard S. (2010). Ethnographys capacity to contribute to the cumulation of theory: a case study of Strong's work on Goffman. J. Contemp. Ethnograph. 39, 421–440. 10.1177/0891241610366710

[B66] Hymes D. (1984). On Erving Goffman. Theory Soc. 13, 621–631. 10.1007/BF00160910

[B67] Jacobsen M. H. (2009). “Goffman through the looking glass: from classical to contemporary Goffman,” in The Contemporary Goffman, ed M. H. Jacobsen (London: Routledge), 1–47.

[B68] Jacobsen M. H. Kristiansen S. (2015). The Social Thought of Erving Goffman. London: Sage.

[B69] Jenkins R. (2008). Erving Goffman: a major theorist of power? J. Power 7, 157–168. 10.1080/17540290802227577

[B70] Lemert C. (2003). “Goffmans Enigma,” in Goffmans Legacy, ed A. J. Trevino, (Lanham: Rowman/Littlefield), xi-xvii.

[B71] Lemert C. Branaman A. (1997). The Goffman Reader. Oxford: Blackwell.

[B72] Lofland J. (1984). Erving Goffman's sociological legacies. Urban Life 13, 7–34. 10.1177/0098303984013001002

[B73] Luhmann N. (1979). Trust and Power. New York, NY: Wiley.

[B74] MacCannell D. (1983). Erving Goffman (1922-1982). Semiotica 45, 1–33. 10.1515/semi.1983.45.1-2.1

[B75] MacIntyre A. (1969). The self as a work of art. New Statesman 28, 447–448. 10.1080/00043249.1969.10793948

[B76] Manning P. (1980). “Goffman's framing order: style as structure,” in The View from Goffman, ed J. Ditton (London: Macmillan), 252–284.

[B77] Manning P. (1989). Goffman's revisions. Philosoph. Soc. Sci. 19, 341–343. 10.1177/004839318901900305

[B78] Manning P. (1991). Drama as life: the significance of Goffman's changing use of the theatrical metaphor. Sociol. Theory 9, 70–86. 10.2307/201874

[B79] Manning P. (1992). Erving Goffman and Modern Sociology. Stanford, CA: Stanford University Press.

[B80] Manning P. (1993). Erving Goffman and Modern Sociology. Stanford: Stanford University Press.

[B81] Manning P. (2000). Credibility, agency, and the interaction order. Symbolic Interact. 23, 283–297. 10.1525/si.2000.23.3.283

[B82] Manning P. (2016a). Goffman and empirical research. Symbolic Interact. 39, 143–152. 10.1002/symb.220

[B83] Manning P. (2016b). The evolution of the concept of social action: Parsons and Goffman. UNLV Gam. Res. Rev. J. 20, 87–96. Available online at: https://digitalscholarship.unlv.edu/grrj/vol20/iss1/6

[B84] Manning P. (2020). “Erving Goffman and dramaturgical sociology” in The Cambridge Handbook of Social Theory, ed P. Kivisto (Cambridge: Cambridge University Press), 226–249. 10.1017/9781316677445.013

[B85] Marx G. T. (1984). Role models and role distance: a remembrance of Erving Goffman. Theory Soc. 13, 649–662. 10.1007/BF00160912

[B86] Menand L. (2009). Some frames for Goffman. Soc. Psychol. Q. 72, 296–299. 10.1177/019027250907200403

[B87] Miller D. L. (1982). Ritual in the work of Durkheim and Goffman: the link between the macro and the micro. Human. Soc. 6, 122–134. 10.1177/016059768200600203

[B88] Morawski J. (2014). Livelihoods of theory: the case of Goffman's early theory of the self. Theory Psychol. 24, 281–304. 10.1177/0959354313520359

[B89] Pallotti G. (2007). “Conversation analysis: methodology, machinery and application to specific settings,” in Conversation Analysis and Language for Specific Purposes, eds H. Bowles and P. Seedhouse (Bern: Peter Lang), 1–31.

[B90] Persson A. (2019). Framing Social Interaction Continuities and Cracks in Goffmans Frame Analysis. London: Routledge.

[B91] Posner J. (2000). “Erving Goffman: his presentation of self,” in Erving Goffman, eds G. A. Manning and G. Smith, vol. I (London: Sage), 99–113.

[B92] Psathas G. (1977). Goffman's image of man. Hum. Soc. 1, 84–94.

[B93] Psathas G. (1996). Theoretical perspectives on Goffman: critique and commentary. Sociol. Perspect. 39, 383–391. 10.2307/1389253

[B94] Ranci F. (2021). The unfinished business of Erving Goffman. Am. Sociol. 52, 390–419. 10.1007/s12108-021-09489-x 34024910 PMC8132032

[B95] Rawls A. W. (1987). The interaction order *sui generis*: Goffman's contribution to social theory. Sociologic. Theory 5, 136–149. 10.2307/201935

[B96] Rawls A. W. (2022). Situating Goffman's “interaction orders” in Durkheim's social facts lineage. Etnografia e Ricerca Qualitativa 1, 27–62. 10.3240/103744

[B97] Riggins S. H. (1990) Beyond Goffman: Studies on Communication, Institution and Social Interaction. Berlin: de Gruyter.

[B98] Rogers M. F. (1977). Goffman on power. Am. Sociol. 12, 88–95.

[B99] Rogers M. F. (2003). “The Personal is dramaturgical (and political): the legacy of Erving Goffman,” in Goffmans Legacy, ed A. J. Treviño (New York: Rowman/Littlefield), 65–75.

[B100] Sacks H. (1984). “Notes on methodology,” in Structures of Social Action, ed J. M. Atkinson, J. Heritage (Cambridge: Cambridge University Press).

[B101] Scheff T. J. (2003). “The Goffman legacy: deconstructing/reconstructing social science,” in Goffman's Legacy, ed A. J. Treviño (New York, NY: Rowman/Littlefield), 50–64.

[B102] Scheff T. J. (2006). Goffman Unbound! A New Paradigm for Social Science. Boulder: Paradigm.

[B103] Schegloff E. A. (1988). “Goffman and the analysis of conversation,” in Erving Goffman—Exploring the Interaction Order, eds P. Drew, A. Wootton (Cambridge: Polity), 89–135.

[B104] Shalin D. N. (2013). Interfacing biography, theory and history: the case of Erving Goffman. Symbolic Interact. 37, 2–40. 10.1002/symb.82

[B105] Sharrock W. W. (1999). “The omnipotence of the actor,” in Goffman and Social Organization, ed G. Smith (London: Routledge), 119–137.

[B106] Smith G. (1988). The sociology of Erving Goffman. Social Stud. Rev. 3, 118–122.

[B107] Smith G. (1989). Snapshots “sub-specie aeternitatis”: Simmel, Goffman and formal sociology. Hum. Stud. 12, 19–57. 10.1007/BF00142838

[B108] Smith G. (1999). “Introduction: interpreting Goffman's sociological legacy,” in Goffman and Social Organization, ed G. Smith (London: Routledge).

[B109] Smith G. (2006). Erving Goffman. London: Routledge.

[B110] Strong P. M. (1988). “Minor courtesies and macro structures,” in Erving Goffman—Exploring the Interaction Order, eds P. Drew, A. Wootton (Cambridge: Polity), 228–249 (originally published in 1983).

[B111] Strong P. M. (2013). Review essay: the importance of being Erving-Erving Goffman, 1922 to 1982. Symbol. Interact. 37, 145–154. 10.1002/symb.80 10316051

[B112] Swedberg R. (2016). Before theory comes theorizing, or how to make social science more interesting. Br. J. Sociol. 67, 5–22. 10.1111/1468-4446.12184 26901757

[B113] Treviño A. J. (2003). “Introduction: Erving Goffman and the interaction order,” in Goffmans Legacy, ed A. J. Treviño (New York: Rowman/Littlefield), 15–49.

[B114] Turner J. H. (2002). Face to Face: Toward a Sociological Theory of Interpersonal Behavior, Stanford: Stanford University Press.

[B115] Verhoeven J. C. (1993). An interview with Erving Goffman, 1980. Res. Lang. Soc. Interact. 26, 317–348. 10.1207/s15327973rlsi2603_5

[B116] Vester H.-G. (1989). Erving Goffman's sociology as a semiotics of postmodern culture. Semiótica 76, 191–203. 10.1515/semi.1989.76.3-4.191

[B117] West C. (1996). Goffman in feminist perspective. Sociologic. Perspectiv. 39, 353–370. 10.2307/1389251

[B118] Wexler M. N. (1984). The enigma of Goffman's sociology. Q. J. Ideol. 8, 40–50.

[B119] Williams R. (1983). Sociological tropes: a tribute to Erving Goffman. Theory Cult. Soc. 2, 99–102. 10.1177/0263276483002001009

[B120] Williams R. (1988). “Understanding Goffman's methods,” in Erving Goffman—Exploring the Interaction Order, eds P. Drew, A. Wootton (Boston: Northeastern University Press).

[B121] Williams S. (1986). Appraising Goffman. Br. J. Sociol. 37, 348–336. 10.2307/590645

[B122] Winkin Y. (1999). “Erving Goffman: what a life? the uneasy making of an intellectual biography,” in Goffman and Social Organization: Studies in a Sociological Legacy, ed G. Smith (London: Routledge), 19–41.

[B123] Winkin Y. Leeds-Hurwitz W. (2013). Erving Goffman: A Critical Introduction to Media and Communication Theory. New York, NY: Peter Lang.

[B124] Wrong D. (1990). “Imagining the real,” in Authors of Their Own Lives, ed B. Berger (Berkeley: University of California Press).

